# Readmissions to General ICUs in a Geographic Area of Poland Are Seemingly Associated with Better Outcomes

**DOI:** 10.3390/ijerph17020565

**Published:** 2020-01-16

**Authors:** Marek Grochla, Wojciech Saucha, Daniel Ciesla, Piotr Knapik

**Affiliations:** 1 Department of Anaesthesiology, Intensive Therapy and Emergency Medicine, Silesian Centre for Heart Diseases, Medical University of Silesia, 41-800 Zabrze, Poland; marek685@op.pl; 2 Department of Science, Education and New Medical Technologies, Silesian Centre for Heart Diseases, 41-800 Zabrze, Poland; w.saucha@sccs.pl (W.S.); d.ciesla@sccs.pl (D.C.)

**Keywords:** ICU, admission, readmission, mortality

## Abstract

Background: Various factors can contribute to high mortality rates in intensive care units (ICUs). Here, we intended to define a population of patients readmitted to general ICUs in Poland and to identify independent predictors of ICU readmission. Methods: Data derived from adult ICU admissions from the Silesian region of Poland were analyzed. First-time ICU readmissions (≤30 days from ICU discharge after index admissions) were compared with first-time ICU admissions. Pre-admission and admission variables that independently influenced the need for ICU readmission were identified. Results: Among the 21,495 ICU admissions, 839 were first-time readmissions (3.9%). Patients readmitted to the ICU had lower mean APACHE II (21.2 ± 8.0 vs. 23.2 ± 8.8, *p* < 0.001) and TISS-28 scores (33.7 ± 7.4 vs. 35.2 ± 7.8, *p* < 0.001) in the initial 24 h following ICU admission, compared to first-time admissions. ICU readmissions were associated with lower mortality vs. first-time admissions (39.2% vs. 44.3%, *p* = 0.004). Independent predictors for ICU readmission included the admission from a surgical ward (among admission sources), chronic respiratory failure, cachexia, previous stroke, chronic neurological diseases (among co-morbidities), and multiple trauma or infection (among primary reasons for ICU admission). Conclusions: High mortality associated with first-time ICU admissions is associated with a lower mortality rate during ICU readmissions.

## 1. Introduction

Readmission to an intensive care unit (ICU) is perceived to be associated with high mortality rates. As a result, ICU readmission rates are currently recognized as ICU performance metrics and quality improvement targets [1,2,3]. This relationship, however, is not observed in crowded emergency departments, where patients who were discharged and then readmitted during a return visit had lower in-hospital mortality and ICU admission rates [4].

Resources for critical care are limited and expensive; appropriate utilization of these resources is therefore essential [5]. Results of recent studies indicate that ICU mortality in Poland is higher than in other European countries [6,7]; however, this is not due to inferior quality of care [8] and is appropriate for the patients’ condition upon ICU admission as assessed using the APACHE II and SAPS III scoring systems [9]. This phenomenon may be primarily associated with the liberal ICU admission policy for patients with a limited chance of survival; however, no research has yet been conducted in Poland to address this issue. The various factors that may contribute to high mortality in Polish ICUs need to be explored.

ICU readmissions have never been investigated in Poland. The only available data come from specialized ICUs admitting patients exclusively after cardiac surgery, in which the mortality rate is generally low and comparable to that observed in other countries [10,11]. We hypothesized that in view of high mortality following initial admission, ICU readmissions are not necessarily associated with a significant increase in mortality and may not indicate an inferior quality of care. 

The aims of this study were to identify a population of patients readmitted to general ICUs in Poland, to compare their course of treatment with the remaining population, and to identify independent predictors of ICU readmission.

## 2. Material and Methods 

This retrospective, multicenter study was performed in general (multidisciplinary) ICUs in the Silesian region of Poland. There are 37 general ICUs operating in this geographical area of Poland, offering a total of 257 ICU beds. Data were derived from the Silesian Registry of Intensive Care Units (“the registry”). This registry is voluntary. Data are entered into the registry by ICU physicians (or medical secretaries working directly under the supervision of ICU physicians). Approximately 35% of all Silesian ICUs report data to the registry, which has been in operation since October 2010. 

The Silesian ICU Registry gathers information on a patient’s general condition and health burden upon admission, the causes of admission, the treatment process, and patient outcomes. All data entered into the registry are verified by the administrator of the database for internal coherence in order to eliminate errors during entry (i.e., conflicting data regarding the same hospitalization). Definitions of all terms used in the registry are available to registered users on the registry website. The entered data, however, are not audited by the registry administrators with regards to their conformity with medical records. It was assumed that such verification should be conducted at the department level. A comprehensive description of the methodology and structure of the Silesian ICU registry has already been described [12]. The registry does not contain any personal data; therefore, the term “patient” is avoided and, if present, it connotes “hospitalization” or “ICU admission” in the whole text of the manuscript. Because this was a retrospective study and the data were entirely anonymous, the Ethical Committee at the Medical University of Silesia in Katowice waived the requirement for patient consent to participate in the study. 

Data from 21,925 ICU admissions from October 2010 to September 2017 were analyzed. In total, 439 admissions were excluded from the analysis. Among them, there were: third and further ICU admissions (*n* = 151), ICU readmissions taking place >30 days after ICU discharge (*n* = 273), and ICU admissions or readmissions with incomplete data (*n* = 6). Ultimately, 21,495 admissions (20,656 first-time ICU admissions and 839 first-time ICU readmissions) were included in the analysis. 

The data from early (≤30 days from ICU discharge) first-time ICU readmissions were compared with first-time ICU admissions. The comparisons included demographic parameters, co-morbidities, the primary reasons for ICU admission, the severity of the condition upon ICU admission, the methods of treatment, and the clinical outcomes. From this, pre-admission and admission variables that independently influenced readmissions were identified. 

Analyses and graphs were generated using the Dell Statistica v13 (Round Rock, TX, USA) data analysis software system. Descriptive statistical methods were used to present demographic data. Quantitative variables were tested for distribution normality using the Kolmogorov-Smirnov test followed by statistical analysis using the two-sided Student’s *t*-test or the Mann-Whitney test. Chi-square analysis with a Yates correction was used for the comparison of qualitative variables. 

**Figure 1:**
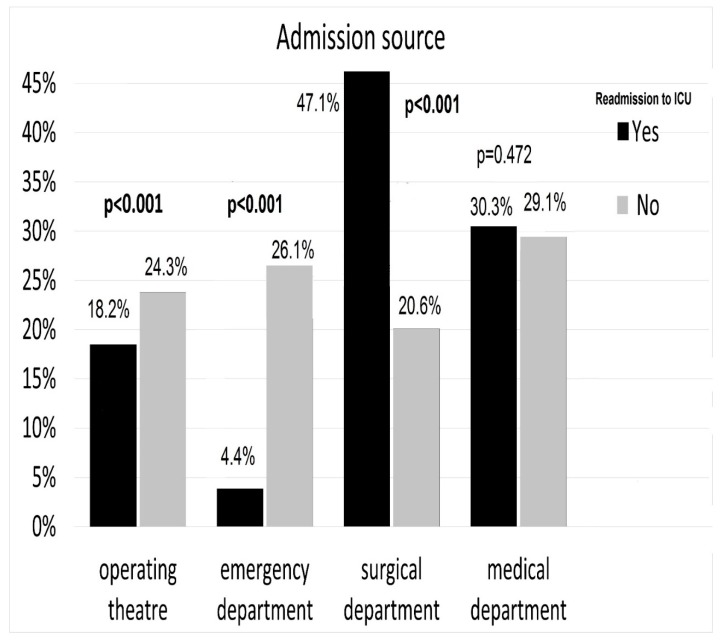
Source of ICU admission.

All variables available in the registry-medical status on ICU admission, primary reason for ICU admission, and admission source—were included in the model. All these variables are presented in Table 1 and Table 2 and Figure 1. The effect of independent variables on the outcome variable of interest (ICU readmission) was calculated using univariate logistic regression. Variables significant in univariate analysis (*p* < 0.05) were included in the multivariate logistic regression analysis. The multivariable model was fitted using a stepwise method where *p* < 0.05 was set as the inclusion and removal criterion. Statistical significance was defined as *p* < 0.05 for all calculations.

## 3. Results

Among the 21,495 ICU hospitalizations recorded in the Silesian ICU registry, 839 were first-time readmissions (3.9%). Of these, 764 ICU readmissions (91.1%) took place within the first two weeks following the previous discharge. 

Readmitted patients were most often admitted to ICUs from the surgical ward (Figure 1). Age and percentage of women among readmitted and non-readmitted patients was similar (63.5 ± 15.6 vs. 64.2 ± 15.7 years, *p* = 0.212 and 39.8% vs. 42.1%, *p* = 0.204, respectively) and readmitted patients stayed longer in the hospital before admission to ICU (14.6 ± 18.4 vs. 4.3 ± 9.3 days, *p* < 0.001). The population admitted to the ICU for the second time had a lower mean admission APACHE II score in comparison to patients admitted for the first time (21.2 ± 8.0 vs. 23.2 ± 8.8, *p* < 0.001). Readmitted patients were also more likely to have chronic lung failure, cachexia, previous stroke, and chronic neurological diseases, but less likely to have a diagnosis of cancer. A comparison of the medical status of both groups during ICU admission is shown in . Moreover, the distribution of the primary causes for ICU admission were different between both groups. Readmitted patients were more likely to be admitted to the ICU due to multiple trauma or infection, but less likely to be admitted due to acute intoxication (Table 2). 

The severity of therapeutic interventions within the first 24 h following ICU admission was lower among readmitted patients in comparison to first-time admissions (TISS-28 score, 33.7 ± 7.4 vs. 35.2 ± 7.8, *p* < 0.001). These patients were also less frequently in shock at the time of ICU admission (25.4% vs. 30.9%, *p* = 0.001). The use of inotropic agents and invasive ventilation was less frequent among readmitted patients during their ICU stay, while percutaneous or surgical tracheostomy was performed more often (25.9% vs. 16%, *p* < 0.001). Readmitted patients were hospitalized for longer in the ICU versus first-time admissions (14.3 ± 19.8 vs. 10.2 ± 17.5 days, *p* < 0.001). A comparison of various therapeutic interventions used during ICU treatment in both groups is shown in Table 3.

Regarding patient outcomes, it was found that readmitted patients were discharged from the ICU in a similar neurological condition according to the Glasgow Outcome Score. The only difference in neurological conditions occurred in the frequency of patients with severe disability (10.7% vs. 7.1%, *p* < 0.001). ICU mortality was lower during the second ICU stay in comparison with the first ICU stay (39.2% vs. 44.3%, *p* = 0.004, Table 4).

**Figure 2:**
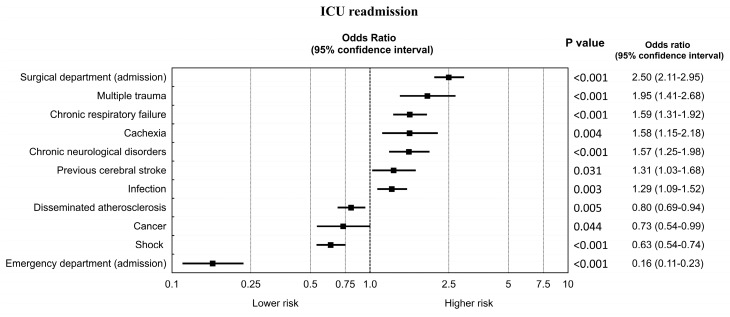
Independent predictors of ICU readmission among patients in the Silesian ICU Registry.

Multivariable analyses conducted based on data presented in Table 1 and Table 2 and Figure 2 indicated that independent predictors for ICU readmission include: admission from the surgical ward (among admission sources), chronic respiratory failure, cachexia, previous stroke, chronic neurological diseases (among co-morbidities), and multiple trauma or infection (among primary reasons for ICU admission). The full list of independent predictors is shown in Figure 2.

## 4. Discussion

To date, no study in Poland has examined factors influencing readmissions to general ICUs. Poland also lacks a nationwide ICU registry. This deficit is partially filled, however, by the presence of a local registry operating in the Silesian region of Poland since October 2010. The Silesian region is a post-industrial area covering only 3.9% of Polish territory, but it is inhabited by 11.9% of the Polish population [13]. Thus, the Silesian ICU registry enables us to extrapolate certain results to the general Polish population. The registry is voluntary and approximately 35% of ICUs operating in this region participate. Furthermore, the collected data have already proved sufficient for gathering information on important trends and patterns [9,13,14]. The issue of the integrity of this local registry is important to note because the data for potential comparisons to our research typically come from large national registries [2,3,15,16].

As mentioned, mortality rates in Polish ICUs are higher than in other European countries [8]. Based on data from the National Institute of Public Health—National Institute of Hygiene (the main institution responsible for monitoring public health in Poland), the 2012 ICU mortality rate was reported to be 42% [7]. This study was based on mandatory, administrative data (which resulted in major methodological limitations), but the observed mortality rate is entirely in agreement with the mortality in the Silesian region of Poland (44.1%). In other countries, however, ICU mortality is much lower and rarely exceeds 20% [3,6,7,15,17,18]. Generally, mortality rates among readmitted patients are significantly higher, exceeding the mortality for index ICU admissions by a large margin. A recent meta-analysis indicated that ICU mortality among readmitted patients across different studies ranged from 10% to 50%, while in the remaining ICU population this figure ranged from 1% to 8% [17]. Studies published by Kramer et al. and Ponzoni et al. have shown that the differences in mortality between readmitted and non-readmitted ICU patients were 21.3% vs. 3.6% and 25.4% vs. 6.4%, respectively [2,19].

In a study published in 2015, Adamski et al. carried out a direct comparison of ICU structure and functioning in two selected ICUs: in Poland and Finland [20]. The difference between ICU mortality rates was significant (41.5% in Poland vs. 10.2% in Finland; *p* = 0.0001). However, the difference between overall hospital mortality rates was smaller (44.7% vs. 21.8%; *p* = 0.0001). Based on these results, it may be calculated that hospital mortality rates among those who left the ICU were only 3.2% in Poland and as much as 11.6% in Finland [20]. 

There are several reasons that could account for the disparity in mortality rates. First, the system of advanced directives signed by the patient before hospital (or ICU) admission is almost unknown in Poland [7]. Second, rapid response teams have not been implemented in the majority of Polish hospitals [20]. Third, there is an increasing fear of legal consequences in cases of either ICU admission refusal or care limitation with do-not-resuscitate orders [8], despite the fact that the official Polish Society guidelines on futile therapy were published a few years ago [21]. Fourth, there is extreme pressure on ICU admissions from the other departments and the hospital administration, mainly because an intermediate care level is poorly developed and poorly funded [22]. Fifth, ICU hospitalizations for patients with TISS-28 scores less than 19 points are avoided if possible because the National Health Fund (NFZ) does not pay for the ICU stays of such patients [23]. As a result, Polish ICUs are forced to admit many patients not receiving any benefit from this level of care (i.e., those too ill for ICU care). This combination of factors may have a profound effect on ICU mortality. It is difficult to estimate the contribution of each factor, and the underlying causes of the high ICU mortality rates, without the creation of a well-designed national medical registry [7]. It seems, however, that we already have enough evidence to start some administrative and educational corrective actions. 

The structure and functioning of critical care services in Poland has already been shown to differ greatly when compared to other countries. This could also account for why data regarding ICU readmissions are different from other countries and can even contradict data from the medical literature. 

The percentage of ICU readmissions in our study was 3.9%. This figure was lower than the mean value of 5.7% presented in a systematic review by Wong et al., in which 32,537 ICU readmissions were analyzed [17]. It was also lower in comparison with the data from the Dutch National Intensive Care Evaluation (NICE) registry, in which a readmission rate of 8.2% was noted among 42,040 ICU admissions [15]. Additionally, Polish patients were generally hospitalized for much longer in the ICU in comparison with other countries [15,24,25]. These findings are not surprising. In many Polish hospitals, there is a lack of step-down units. This deficit is likely associated with cautious patient discharge from the ICU and may result in a lower percentage of ICU readmissions combined with longer mean ICU stays. 

In studies concerning ICU readmissions, the influence of older age has been emphasized as a predisposing factor [16,17,26]. In Poland, however, patients readmitted to the ICU were found not to differ from their non-readmitted counterparts in terms of age. Paradoxically, this was probably due to the poor medical condition of Polish patients at the time of index ICU admission combined with the high mortality rate during the index ICU stay. It should be taken into account that those discharged from Polish ICUs constitute only 55%–60% of initially admitted patients (rather than the vast majority of patients, which is often observed in other countries). 

Regarding co-morbidities, readmitted patients in Poland were found to be more frequently diagnosed with chronic respiratory failure and cachexia. They were also more likely to suffer from chronic lung diseases, prior stroke, and chronic neurological disorders, and less likely to have a diagnosis of cancer. Studies from the literature containing comparisons of readmitted and non-readmitted patients present slightly different results regarding co-morbidities. In contrast to our findings, readmitted patients in the literature were more likely to have a diagnosis of cancer, but the frequency of chronic neurological disorders was similar in both groups [19,25]. Similar to our results, readmitted patients were also more likely to suffer from chronic lung diseases [19,25]. 

Readmitted patients in our study had a lower mean admission APACHE II score (21.2 ± 8.0 vs. 23.2 ± 8.8) and a lower mean TISS-28 score in the first 24 h of their ICU stay (33.7 ± 7.4 vs. 35.2 ±7.8) in comparison to the first-admission patients. Opposite results can be found in the literature, with APACHE II scores found to be higher among readmitted patients in at least two previous studies [27,28]. Our findings were therefore unexpected, and again may stem from the fact that patients discharged from the initial ICU stay constituted only 55%–60% of the initially admitted patients. 

The distribution of the primary reasons for ICU admission among readmission and index admissions in our study could be linked to the previously presented differences in their APACHE II and TISS-28 scores at admission. A lower percentage of readmitted patients were in shock at the time of admission. Additionally, the infusion of vasopressors and invasive ventilation was less frequent during the ICU stay of these patients. These proportions were again unexpected. Ponzoni et al. reported that readmitted patients in Brazil required both vasopressors and invasive ventilation more frequently (41.8% vs. 25.9%; *p* < 0.001 and 35.6% vs. 23.7%; *p* < 0.001) [19]. More importantly, the need for vasopressors was also higher among readmitted patients in a population of 682,975 patients admitted to 262 ICUs in the UK between 2010 and 2014 [3]. Woldhek et al. [24], however, found that readmitted patients in the Netherlands were less frequently intubated and mechanically ventilated (77.4% vs. 87.5%; *p* < 0.001). 

Patients hospitalized in Polish ICUs were more frequently admitted due to exacerbation of chronic respiratory failure, polytrauma, and infection without sepsis. In the literature, patients were more commonly readmitted when they were elderly and had pneumonia, heart failure or sepsis [16,25,26].

Readmitted patients stayed significantly longer in the ICU compared with first-time admissions (14.3 ± 19.8 vs. 10.2 ± 17.5 days, *p* < 0.001). These proportions are in line with data from the literature [19,29,30]. Moreover, the lower mortality observed in our study among patients readmitted to the ICU may be associated with the longer ICU stay. It follows that ICU admission for a very severe condition with little chance of survival may result in a very short ICU stay. Furthermore, the condition of Polish patients at the time of ICU discharge needs to be examined. The data in Table 4 clearly indicate that around 55% of patients (in both groups) could be categorized as “unfavorable outcome at hospital discharge” (death, minimally conscious/vegetative state, or severe disability). It seems, therefore, that there is a serious systemic error in the structure of our ICU admissions, with a profound impact on the functioning of at least 350 general ICUs treating at least 50,000 patients per annum [7].

Independent predictors for ICU readmission in Poland included admission from the surgical ward (among admission sources), chronic respiratory failure, cachexia, previous stroke, chronic neurological diseases (among co-morbidities), and multiple trauma or infection (among primary reasons of ICU admission). It is difficult to explain these findings as they may be specific to the Polish ICU population. Independent predictors for ICU readmission identified elsewhere are variable and do not demonstrate any consistent relationship [25,26]. 

There are important limitations to the current study. First, the sample is limited because the registry is both local (Silesian region only) and voluntary (only 35% of Silesian ICUs report to the Silesian ICU Registry). Second, the registry does not contain personal data, therefore it was impossible to investigate the characteristics and clinical course of patients readmitted to the ICU during their index ICU stay. By protecting patients’ personal data, this system does not permit any kind of long-term survival study or follow-up analysis. Third, some terms used in the registry have not been precisely defined (e.g., general status at ICU discharge). Fourth, the exact time of readmission has not been precisely defined in the Silesian ICU registry. Instead, only time intervals have been used (such as readmission below 14 days, 14–30 days, and above 30 days from the time of ICU discharge). This is a critical issue because the European Society of Intensive Care Medicine advocates for using ICU readmission within two days of discharge as a quality indicator, as readmissions occurring later may represent events unrelated to the ICU level of care [3]. In addition, we also had no data regarding the inter- and intra-variability among ICU staff collecting and entering data into the registry. All these deficiencies, however, are balanced by the large sample size and the significance of the data. 

## 5. Conclusions

In conclusion, general ICUs in Poland are characterized by high mortality and relatively low readmission rates. Surprisingly, clinical outcomes among patients readmitted to the ICU are better in comparison to first-time ICU admissions. This finding, however, should be approached with caution. High mortality during first-time ICU admissions may result in seemingly better outcomes when analyzing ICU readmissions.

## Figures and Tables

**Table 1:** Medical status on ICU admission.

Group of Variables		Readmitted		Non-Readmitted		*p*
		(*n* = 839)		*(n* = 20.656)		
**Co-morbidities**	Coronary artery disease	373	(44.5%)	8600	(41.6%)	0.112
	Heart failure	308	(36.7%)	7247	(35.1%)	0.352
	Arterial hypertension	415	(49,5%)	10.548	(51.1%)	0.382
	Disseminated atherosclerosis	266	(31.7%)	7046	(34.1%)	0.160
	Chronic respiratory failure	144	(17.2%)	2435	(11.8%)	**<0.001**
	Home oxygen therapy	14	(1.7%)	274	(1.3%)	0.489
	Extreme obesity	50	(6.0%)	1131	(5.5%)	0.599
	Cachexia	45	(5.4%)	719	(3.5%)	0.005
	Alcoholism	62	(7.4%)	1920	(9.3%)	0.070
	Diabetes	209	(24.9%)	5098	(24.7%)	0.912
	Chronic renal failure	117	(14.0%)	2996	(14.5%)	0.688
	Dialysis dependency	12	(1.4%)	257	(1.2%)	0.751
	Previous cerebral stroke	79	(9.4%)	1488	(7.2%)	**0.019**
	Chronic neurological disorders	93	(11.1%)	1544	(7.5%)	**<0.001**
	Systemic autoimmune diseases	9	(1.1%)	244	(1.2%)	0.902
	Post-transplant	2	(0.2%)	45	(0.2%)	0.801
	Cancer	48	(5.7%)	1594	(7.7%)	0.039
	Pregnancy	0	(0.0%)	40	(0.2%)	0.386
	None	68	(8.1%)	2029	(9.8%)	0.113

Bold is when *p* < 0.05.

**Table 2:** Primary reason of ICU admission.

Primary Reason of ICU Admission	Readmitted			Non-Readmitted		*p*
	(*n* = 839)			(*n* = 20.656)		
Shock	213	(25.4%)	6375		(30.9%)	**0.001**
Cardiac arrest	186	(22.2%)	5168		(25.0%)	0.067
Postoperative	269	(32.1%)	6157		(29.8%)	0.174
Multiple trauma	48	(5.7%)	765		(3.7%)	**0.004**
Craniocerebral trauma	45	(5.4%)	1000		(4.8%)	0.543
Acute pancreatitis	15	(1.8%)	320		(1.6%)	0.686
Obstetric complications	1	(0.1%)	75		(0.4%)	0.384
Acute neurological disorders	57	(6.8%)	1572		(7.6%)	0.418
Intoxication	4	(0.5%)	337		(1.6%)	**0.013**
Severe metabolic disorders	35	(4.2%)	1148		(5.6%)	0.099
Sepsis	62	(7.4%)	1448		(7.0%)	0.724
Infection	203	(24.2%)	3802		(18.4%)	**<0.001**
Advanced monitoring	447	(53.3%)	11.317		(55.6%)	0.409

Bold is when *p* < 0.05.

**Table 3:** ICU treatment.

ICU Treatment	Readmitted		Non-Readmitted		*p*
	(*n* = 839)		(*n* = 20.656)		
Catecholamines	580	(69.1%)	14.992	(72.6%)	**0.031**
Intubation	476	(56.7%)	13.333	(64.6%)	**<0.001**
Tracheostomy	217	(25.9%)	3306	(16.0%)	**<0.001**
Renal replacement therapy	73	(8.7%)	1938	(9.4%)	0.546
Operation while in the ICU	86	(10.3%)	1866	(9.0%)	0.254
Intra-aortic balloon pump	20	(2.4%)	537	(2.6%)	0.783
ECMO	5	(0.6%)	58	(0.3%)	0.184

Bold is when *p* < 0.05.

**Table 4:** Discharge and outcome.

Discharge and Outcome		Readmitted		Non-Readmitted		*p*
		(*n* = 839)		(*n* = 20.656)		
General status at ICU discharge	Good	212	(25.3%)	5577	(27.0%)	0.285
	Average	257	(30.6%)	5135	(24.9%)	**<0.001**
	Severe	41	(4.9%)	800	(3.9%)	0.163
	Death	329	(39.2%)	9144	(44.4%)	**0.004**
Neurological status (Glasgow Outcome Score)	Good	256	(30.5%)	6588	(31.9%)	0.421
	Moderate disability	120	(14.3%)	2558	(12.4%)	0.110
	Severe disability	90	(10.7%)	1465	(7.1%)	**<0.001**
	Minimally conscious or vegetative	44	(5.2%)	901	(4.4%)	0.256
	Death	329	(39.2%)	9144	(44.4%)	**0.004**
Discharge to	Same hospital—another department	328	(39.1%)	8142	(39.4%)	0.879
	Other hospital	140	(16.7%)	2820	(13.7%)	**0.014**
	Long-term facility	20	(2.4%)	215	(1.0%)	**<0.001**
	Home	22	(2.6%)	335	(1.6%)	**0.037**
	Death	329	(39.2%)	9144	(44.4%)	**0.004**

Bold is when *p* < 0.05.

## Abbreviations

The following abbreviations are used in this manuscript:

ICU	Intensive Care Unit
APACHE	Acute Physiology Age Chronic Health Evaluation
SAPS	Simplified Acute Physiology Score
TISS	Therapeutic Intervention Scoring System
